# HLA-G/sHLA-G and HLA-G-Bearing Extracellular Vesicles in Cancers: Potential Role as Biomarkers

**DOI:** 10.3389/fimmu.2021.791535

**Published:** 2021-11-11

**Authors:** Peilong Li, Nan Wang, Yi Zhang, Chuanxin Wang, Lutao Du

**Affiliations:** ^1^ Department of Clinical Laboratory, The Second Hospital, Cheeloo College of Medicine, Shandong University, Jinan, China; ^2^ Shandong Engineering & Technology Research Center for Tumor Marker Detection , Jinan, China; ^3^ Shandong Provincial Clinical Medicine Research Center for Clinical Laboratory, Jinan, China; ^4^ School of Public Health, Shandong First Medical University & Shandong Academy of Medical Sciences, Taian, China; ^5^ Department of Respiratory and Critical Care Medicine, Qilu Hospital, Cheeloo College of Medicine, Shandong University, Jinan, China

**Keywords:** HLA-G, tumor, immune escape, extracellular vesicles, biomarker

## Abstract

As a non-classic major histocompatibility complex (MHC) class I molecule, human leukocyte antigen G (HLA-G) is expressed in fetal-maternal interface and immunoprivileged site only in healthy condition, and in pathological conditions such as cancer, it can be *de novo* expressed. It is now widely accepted that HLA-G is a key molecule in the process of immune escape of cancer cells, which is ubiquitously expressed in the tumor environment. This raises the possibility that it may play an adverse role in tumor immunity. The expression level of HLA-G has been demonstrated to be highly correlated with clinical parameters in many tumors, and its potential significance in the diagnosis and prognosis of cancer has been postulated. However, because HLA-G itself has up to seven different subtypes, and for some subtypes, detected antibodies are few or absent, it is hard to evaluate the actual expression of HLA-G in tumors. In the present work, we described (a) the structure and three main forms of HLA-G, (b) summarized the mechanism of HLA-G in the immune escape of tumor cells, (c) discussed the potential role of HLA-G as a tumor marker, and reviewed (d) the methods for detecting and quantifying HLA-G.

## Introduction

**Figure 1:**
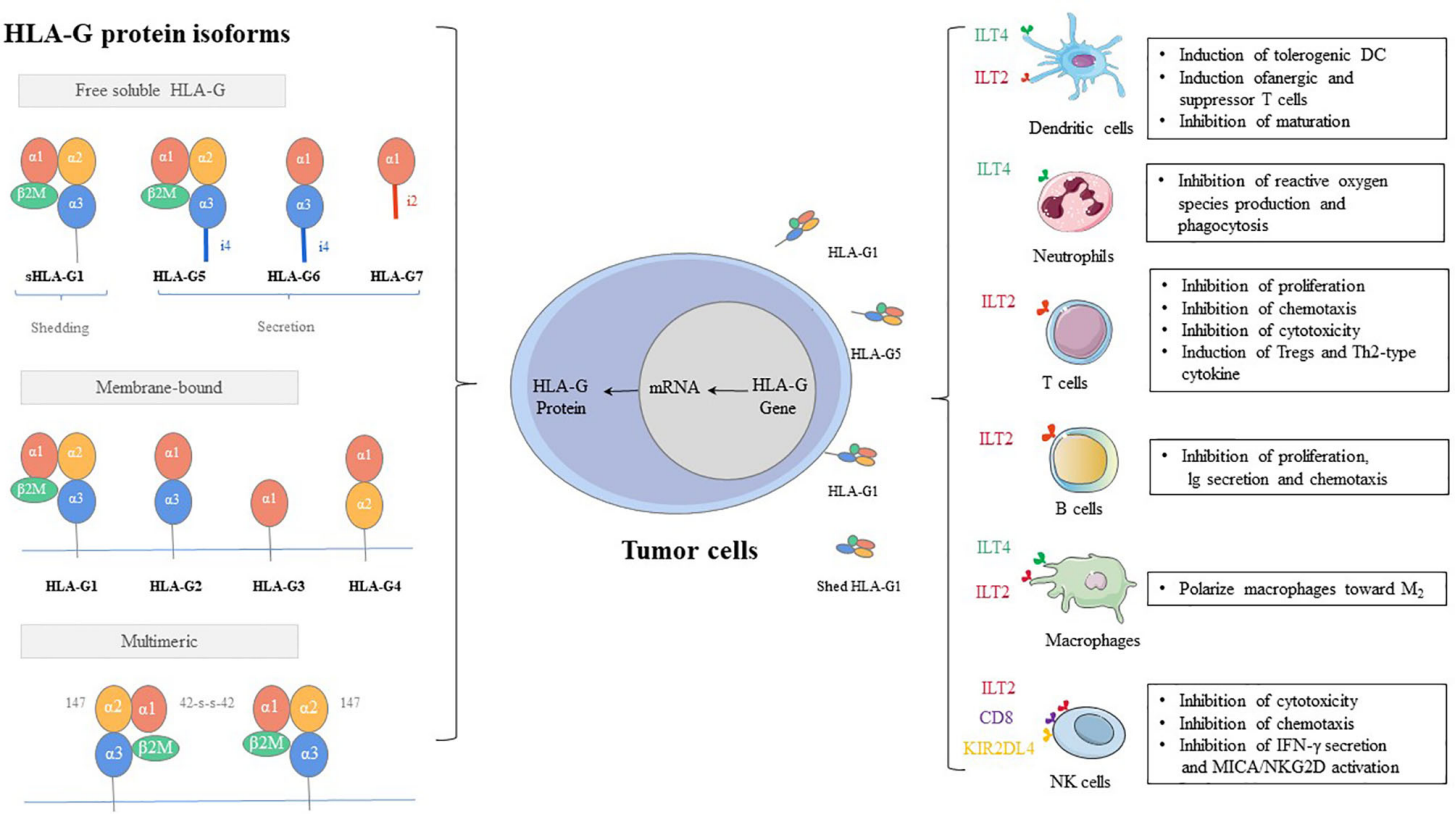
HLA-G protein isoforms and immune-inhibitory function.

As early as 1983, human leukocyte antigen G (HLA-G) is first observed on the cytotrophoblast at the fetal-maternal interface (1). As a class of major histocompatibility complex (MHC) I molecules, HLA-G showed low polymorphic in the coding region, while several polymoprphism have been describe in non-coding region of the locus (3’UTR, and 5’URR regions) (2). The exons and introns of the HLA-G gene are the same as those of classic MHC class molecules, consisting of eight exons and seven introns (3, 4). However, HLA-G shows only limited genetic polymorphism. The main reason can be attributed to that the terminator of HLA-G is located in the second codon of exon 6, and thus most of exon 6 and all of exons 7 and 8 cannot be translated into protein (5, 6). HLA-G can exist in a variety of structures, which can not only be expressed on the cell surface but also exist in the form of secretion (7, 8). There are seven isoforms, which encode four membrane-bound (HLA-G1, -G2, -G3, and -G4) and three soluble (HLA-G5, -G6, and -G7) protein isoforms (9). Each HLA-G subtype contains one to three spherical domains (α1, α2, and α3) encoded by exon 2, exon 3, and exon 4 (**Figure 1**).

HLA-G is initially considered to be useful for fetal establishment and maintenance of maternal-fetal immune tolerance (10, 11). Therefore, initial studies have mainly focused on its role in regulating maternal immune cell responses and protecting the fetus from natural killer (NK) cell-mediated lysis (12–14). Fetal and tumor development are similar and characterized by rapid tissue proliferation, which is associated with the expressions of anti-apoptotic factors and telomerase (14–17). It is worth noting that since immunoregulatory sites can be shared between tumor development and placenta formation, both placenta and tumor are protected by the immune system and resistant to induction by the immune microenvironment. Therefore, the research focus of HLA-G has gradually shifted to tumors (18).

In the malignant environment where tumors occur, the expression of HLA-G in melanoma is first reported in 1998 (19), and then the abnormal expression of HLA-G is observed in a variety of malignant tumors, such as lung cancer (20–28), gastric cancer (29–37), ovarian cancer (OC) (38–47), breast cancer (48–54), and hematopoietic tumor (55–58). With the deepening of research, the expression of HLA-G in solid malignant tumors and its potential clinical relevance have attracted increasing attention (59). Abnormal expression of HLA-G plays a variety of roles in the progression of malignant tumors, such as inducing apoptosis, inhibiting immune cytolysis and cytotoxicity, and chemotaxis of regulatory cells and damage of different immune effector cells through receptor binding and/or trogocytosis (60).

In addition, HLA-G is secreted in a variety of body fluids, as either free soluble HLA-G (sHLA-G) or part of extracellular vesicles (EVs), and it has been extensively studied as tumor markers. Among them, sHLA-G is derived from the secretion of sHLA-G homotypes, such as HLA-G5, HLA-G6, and HLA-G7, and the shedding of membrane-bound HLA-G homotypes, such as HLA-G1, cleaved by proteolytic hydrolysis (61). Soluble isoforms can be detected in saliva (62, 63), ascitic fluid (47), plasma (26, 33, 48, 49, 56, 64–69), thymus (70), seminal plasma (71), cerebrospinal fluid (CSF) (72, 73), human first trimester and term placentas *in situ* and *in vitro* (74), and cell culture supernatant (75, 76). High levels of sHLA-G are correlated with tumor histological type, lymph node metastasis, and patient survival, which can be used as a tumor marker to provide a basis for early diagnosis, differentiation, and prognosis (21). However, it remains largely unclear whether HLA-G-bearing EVs are produced by tumor cells, while there is a functional association between the HLA-G-bearing EVs and various tumors, such as melanoma, breast cancer, and kidney cancer (77). In summary, sHLA-G and HLA-G-bearing EVs may provide unpredictable diagnostic opportunities to monitor tumor status and progression.

## HLA-G: A Key Immune Evasion Molecule in Tumors

**Figure 2:**
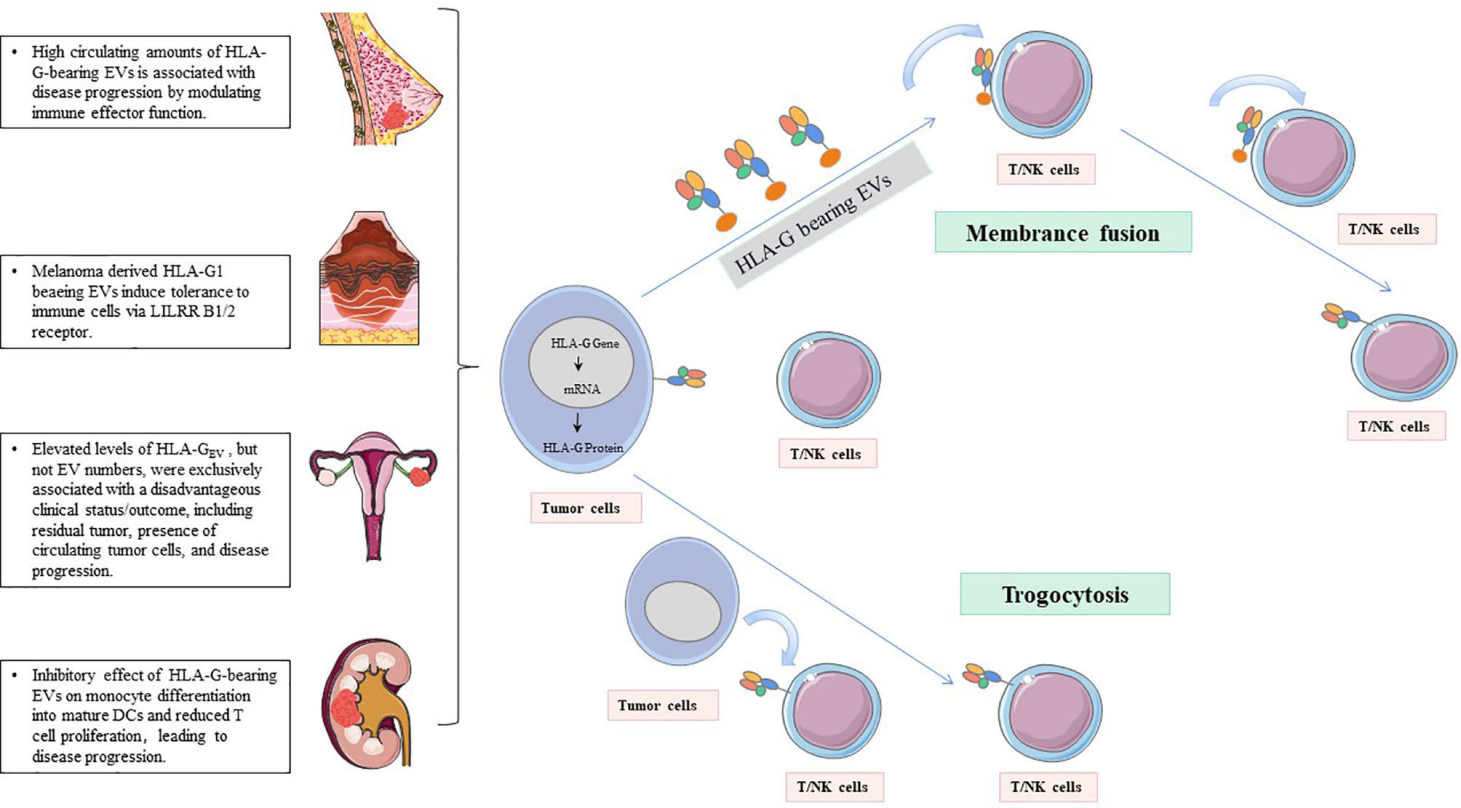
HLA-G-bearing EVs with potential immunological and clinical relevance. NK cells and T cells acquire HLA-G from HLA-G+tumor cells or HLA-G+immune cells *via* the process of trogocytosis and/or EVs.

In cancer, abnormal expression of HLA-G is considered a key strategy of tumor cells to evade immune surveillance, which is strongly supported by the high incidence of tumors in patients treated with immunosuppressive agents after organ and stem cell transplantation (78). The continued construction of tumor phenotypes is thought to be a result of immune-mediated tumor recognition, a phenomenon known as immune editing of cancer. Three stages define the process of immune editing: elimination (immune surveillance), balance (duration/dormancy), and escape (progression) (79, 80). These three stages integrate the immune system’s ability to protect the host from cancer and promote cancer development (81). HLA-G is involved in all three stages, and it is highly necessary to understand the role of HLA-G in tumor immune escape to better develop effective anti-tumor strategies. In the present work, we summarized the main mechanisms of HLA-G-mediated immunosuppression in three aspects as follows:

1. Inhibitory receptors of HLA-G, such as KIR2DL4/CD158d, ILT-2/CD85j, ILT-4/CD85d, CD8, and CD160, can directly exert the immunosuppressive effect by HLA-G. These inhibitory receptors can express in all monocytes, as well as B cells, T cells, and NK cells. Especially, ILT2 receptors are present in subgroups of dendritic cells (DCs) and myeloid-derived suppressive cells (MDSCs) (82–85). ILT4 receptors are mainly expressed in DCs, neutrophils, monocytes, and MDSCs (86–88). KIR2DL4 receptors are mainly expressed in decidual NK cells (89). The mechanisms by which these receptors participate in immunosuppressive effects induced by HLA-G include inhibition of differentiation, cytokine secretion and chemotaxis, immune cell proliferation, cytotoxicity, and induction of MDSCs or M2-type macrophages and regulatory cells (90, 91) (**Figure 1**).
2. MDSCs, regulatory T cells (Tregs), and tolerogenic DCs can participate in immune escape regulated by HLA-G *via* an indirect immunosuppressive way. The sub-population of tolerogenic DCs, DC-10s, expresses a high level of HLA-G and induces adaptive type 1 Tregs (Tr1) through the HLA-G/ILT4 signaling pathway (92). On the other hand, tolerogenic DCs produce CD8+CD28+ and CD4+CD25+CTLA-4+ Tregs under the induction of HLA-G, which further strengthens the ability of immune escape of tumor cells (93).
3. HLA-G uses dynamic transfer mechanisms between cells, such as trogocytosis (membrane-bound HLA-G) and EVs (membrane-bound and sHLA-G) (77, 94–96) (**Figure 2**). Trogocytosis is the process of transporting secretory vesicles or other membrane vesicles from the cell through the cell membrane (97). Activated T cells and NK cells obtain membrane fragments containing functional HLA-G from HLA-G+ or tumor cells through the process of exocytosis. HLA-G-modified cells can immediately reverse immune effector functions to tolerogenic function (98, 99). The generic term EVs are phospholipid bilayer-enclosed vesicles, which are highly heterogeneous in size, molecular content, and membrane composition depending on the state, micro-environment, and the cell of origin (100, 101). According to biogenesis, EVs are characterized by apoptotic bodies (AB) (>500 nm), microvesicles (100-1,000 nm), and exosomes (70-150 nm) (102). In the tumor state, especially in the established acidic microenvironment, EVs can directly fuse with cancer cells, or carry out the transportation and exchange of biologically active substances through endocytosis, phagocytosis, and micropinocytosis, thereby contributing to the intercellular signaling mechanism, providing the tumor with oxygen, metabolites, nutrients and other soluble factors, and making tumor development possible (103).

## Elevated HLA-G in Tumor Patient Tissues as Tumor Indicators

Over the years, many studies have reported that HLA-G is preferentially detected in the primary tumor site and metastatic tumor site, while it is rarely detected in the tumor spontaneous regression site, adjacent tissues, or healthy tissues (104–106). Immunohistochemical (IHC) staining is usually used to detect the expression frequency of HLA-G in tissues, which is combined with clinical results for analysis. In most cancers, the expression of HLA-G is related to the patient’s poor clinical outcome (25, 107, 108).

In liver tissue, HLA-G is detected in the primary site of hepatocellular carcinoma, while its expression is low in benign lesions represented by liver cirrhosis (109). In thyroid tissue, the cell staining efficiency of HLA-G antigen in follicular adenocarcinoma and thyroid cancer is significantly higher compared with the normal thyroid and goiter (110). In ovarian tissue, HLA-G is always co-localized with CA125 protein, indicating that OC cells express HLA-G, while normal cells do not (39). In cervical cancer lesions, the expression of HLA G is an important predictor of CIN I and age, which is not affected by other variables. In addition, HLA-G interacts with immunosuppression induced by human papillomavirus infection, leading to more serious clinical outcomes observed in patients with CIN III and invasive cervical cancer (111, 112).

In oral squamous cell carcinoma (OSCC), according to IHC and reverse transcription-polymerase chain reaction (RT-PCR) results, the higher the TNM stage, the higher the protein expression level of HLA-G, and the histological grade and lymph node metastasis are positively correlated with the expression of HLA-G (107). These results indicate that HLA-G is related to the malignant transformation of tumors, supporting that HLA-G is an indicator of early diagnosis and dynamic monitor. The expression of HLA-G in tissues may also be an important indicator for the prognosis of cancer patients. In 201 colon cancer patients, IHC staining shows that the survival time of patients with HLA-G-positive tumors is significantly shorter compared with those carrying HLA-G-negative tumors (113). In multivariate analysis, HLA-G shows the potential as an independent prognostic factor.

In a joint analysis of HLA class I, HLA-E, and HLA-G to predict the prognosis of colorectal cancer (CRC), three tumor immune phenotypes are generated by comprehensively analyzing the expressions of all markers, resulting in strong immune system tumor recognition, intermediate immune system tumor recognition, and poor immune system tumor recognition. These immune phenotypes represent important and independent clinical prognostic characteristics of colon cancer (108). In nasopharyngeal carcinoma (NPC), HLA-G is positively correlated with CD68+ macrophages and IL-10 expression, indicating that HLA-G may regulate immune escape in NPC (114). Among 522 NPC specimens, the expression of HLA-G at the protein level is detected in 79.2% of cases. In addition, the high expression of HLA-G predicts the low survival rate of NPC patients (114). Moreover, HLA-G is suggested to be an independent predictor of cancers, such as esophageal squamous cell carcinoma (115), gastric cancer (37, 116), breast cancer (52, 117, 118), and OC (38, 44, 119). Finally, the differential expressions of HLA-G can also help predict and diagnose different subtypes. Significant differences between groups have been observed between low-grade glioma and high-grade glioma tissues (120). Besides, the expression of HLA-G in the non-luminal subtypes of invasive ductal carcinoma of the breast is significantly higher compared with the luminal subtypes (121).

## sHLA-G in the Blood of Tumor Patients as Circulating Tumor Markers

In the serum of healthy people, the content of HLAG is 20 ng/mL and significantly lower compared with cancer patients. sHLA-G is produced and secreted mainly by immune cells and tumors (122). For example, in acute leukemia, the level of sHLA-G in T cells and monocytes in the serum is detected by enzyme-linked immunosorbent assay (ELISA), which is averagely five times higher compared with healthy controls. Moreover, sHLA-G can be secreted *in vitro* by DCs, lymphocytes, plasma cells, and monocytes/macrophages (56), and these secreted sHLA-G molecules cause anti-tumor reactions locally in the tumor or along with the circulatory system to the whole body. Next, we described the current status of sHLA-G in the serum of tumor patients as tumor markers from the aspects of diagnosis, prognosis, and identification (**Table 1**).

**Table 1:** Clinical research involving diagnosis and prognosis of sHLA-G in the blood.

Cancer type	Sample source	Sample size	Methods(Ab)	Experimental result evaluation	Expression evaluation of HLA-G	Ref.
**Breast cancer**	plasma	142 (procured before NACT) 154 (after)	ELISA	The total concentration of sHLA-G plasma levels (median [range] ng/mL) from BC patients (n = 102) before (41.3 [4.4–117.6]) and after (44.6 [3.1–117.6]) NACT was significantly increased compared with 16 healthy female controls (16.3 [4.0–37.8]).	The free soluble and vesicular HLA-G as prognostic markers, whereas the total sHLA-G levels without dividing into subcomponents were not related to clinical outcome.	(48)
	plasma	92 (patients) 70 (controls)	ELISA	Concentration of the plasma sHLA-G was with the median of 82.19 U/mL (range 13.50 –191.37) for BC patients, and 9.65 U/mL (range 4.38 – 69.69) for normal controls,	Plasma sHLA-G levels might be a useful preoperative biomarker for diagnosis	(49)
**Ovarian cancer**	plasma	79 (patients) 80 (controls)	ELISA (MEM-G9)	In OC patients, sHLA-G1 levels were more increased than HLA-G5 levels.	As a potential biomarker for advanced and complicated OC.	(66)
**Lung cancer**	plasma	137	ELISA	In lung cancer patients, the plasma levels (median[range]) of sHLA-G were significantly increased compared with healthy controls (34 ng/mL [3.6–160] vs. 14 ng/mL [0–98]).	Plasma levels of sHLA-G is potent predictors for overall survival (OS) in lung cancer patients.	(26)
	plasma	91(patients) 150(controls)	ELISA	The median plasma sHLA-G was 34.0 U/mL (range 3.13 – 275.5) in NSCLC patients and 20.4 U/mL (range 0.97 -270.6) in controls.	HLA-G may be a potential therapeutic target, and plasma sHLA-G of NSCLC patients can be used as a prognostic factor for NSCLC.	(23)
**Colorectal cancer**	plasma	133	ELISA (MEMG/9)	sHLA-G levels were higher in patients with mucinous carcinoma (MC).	A useful prognostic marker and predictive biomarker of therapeutic response in advanced CRC.	(64)
**Endometrial cancer**	plasma	40 (patients) 45 (controls)	ELISA	The majority of EC patients expressed the sHLA-G1 subtype (75%), and only 25% expressed the HLA-G5 isoforms.	Related to clinical progress.	(123)
**Thyroid carcinoma**	plasma	85 (patients) 77 (30 days after surgery)	ELISA (MEM-G/9)	sHLA-G was decreased in patients with invasion.	Associated with tumor invasion.	(65)
**Thyroid carcinoma**	plasma	121 (patients) 183 (underwent PTC surgery)	ELISA (MEM-G9)	sHLA-G level was significantly higher in PTC patients than those without markers of aggressiveness	sHLA-G as a potential novel marker of PTC aggressiveness	(67)
**Esophageal squamous cell carcinoma**	plasma	41 (patients) 153 (controls)	ELISA	The median plasma concentration of sHLA-g in ESCC patients was 152.4 U/mL (range 28.8-239.5) *versus* 8.9 U/mL (range 4.6-63.5) in normal controls.	May be a useful biomarker for preoperative diagnosis.	(115)
**Breast cancer**	serum	80 (patients) 80 (controls)	ELISA	Levels of sHLA-G were higher in the breast cancer group (median117.2 U/mL) compared with the control group (median 10.1 U/mL, P, 0.001).	Measurement of sHLA-G concentrations has diagnostic value for detecting breast cancer and metastasis.	(124)
**Lung cancer**	serum	191 (patients) 191 (controls)	ELISA	The mean serum level of sHLA-G in NSCLC patients (53.3 ± 4.6 U/mL) was significantly increased compared with controls (8.36 ± 0.4 U/mL).	Serum sHLA-G levels in NSCLC patients could be useful biomarkers for the diagnostic and prognosis of NSCLC.	(22)
**Colorectal cancer**	serum	398	ELISA (MEMG/9)	Median sHLA-G was significantly higher in cancer compared with normal CRC, hyperplastic polyps, inflammatory bowel disease, and adenomas (all P < 0.001).	May be a useful indicator in differentiating colorectal cancer from benign colorectal diseases.	(125)
**Thyroid carcinoma**	serum	145	ELISA	sHLA-g in serum was increased in patients with thyroid carcinoma compared with healthy controls (P < 0.05).	Affects the progression of thyroid cancer.	(126)
**Oral squamous cell carcinoma**	serum	216 (patients) 193 (controls)	ELISA	sHLA‐G levels were higher in the OSCC patients than healthy subjects (85.694 ± 69.966 U/mL *vs.* 11.404 ± 10.424 U/mL).	sHLA‐G may act as a promising biomarker for non-invasive diagnosis of OSCC.	(127)
**Head and neck squamous cell carcinoma**	venous blood	383 (patients) 383 (controls)	PCR	Individuals with Del/Ins and Ins/Ins genotypes were at greater risk of HNSCC disease than those with Del/Del genotypes.	The C/C, Del/Ins and Ins/Ins genotypes as well as C and Ins alleles may be the major risk factors for the strong influence of tobacco on HNSCC in Indian.	(128)
	serum	120 (patients) 99 (controls)	ELISA	Compared with the control group (6.45 ± 1.31 ng/L), the levels of SHLA-G in patients were significantly higher (8.25 ± 1.74 ng/L).	Potential diagnostic serum protein markers.	(129)
**Esophageal cancer**	EDTA blood	239 (patients) 467 (controls)	PCR	In the Kazakh region, individuals with the -14 bp/-14 bp and C/C genotypes had a 2.82 times higher risk of developing EC than those with the +14 bp/+14 bp and C/C genotypes.	The 14 bp deletion/insertion of HLA-G gene may play a role in EC susceptibility of Kazakh.	(130)

In terms of diagnosis, sHLA-G is abnormally expressed in the plasma in breast ductal carcinoma (131), head and neck squamous cell carcinoma (129), gastric cancer (33), CRC (125), and papillary thyroid carcinoma (65), which is considered to be a preoperative diagnosis of cancer histopathology potential marker of aggressiveness. It is worth noting that in gastric cancer, researchers have found that sHLA-G in combination with common serum tumor markers, such as CA72-4, CA125, and CA19-1, can improve the clinical screening of gastric cancer compared with sHLA-G alone (132). In patients with breast cancer, the expression of HLA-G is negatively correlated with proliferation factor (133). In addition, the concentration of sHLA-G in plasma helps predict and diagnose cancers of different subtypes. Compared with patients with simple lobular carcinoma and simple ductal carcinoma, patients with mixed lobular lesions and breast ducts have a more significant increase in sHLA-G (131). ELISA also shows that the plasma levels of sHLA-G in patients with liver cancer are higher compared with the control group (134). These results support that sHLA-G can be used for tumor early diagnosis.

In terms of prognosis, the high expression of sHLA-G in patients with non-small cell lung cancer is significantly related to poor overall survival (OS), especially in patients with advanced cancer (135). An interesting phenomenon has been found in the Tunisian population that patients with HLA-G*01:04:01 alleles have elevated plasma levels of sHLA-G, while patients who do not carry HLA G*0105N alleles (cannot encode HLA-G1 protein) express a dramatically reduced level of sHLA-G in plasma (22). In CRC, the prognostic value of plasma sHLA-G is mainly reflected by predicting the risk of liver metastasis in patients with stage II and III CRC. Hauben and colleagues have found that the sHLA-G levels are associated with shorter liver metastasis-free survival (LMFS) in patients with stage II CRC and longer LMFS in patients with stage III CRC. The possible reason can be attributed to the fact that the stage III patients receive chemotherapy before the sample collection, and the tumor cells are damaged (64). Therefore, the differential level of plasma sHLA-G is a predictive biomarker of treatment response to advanced CRC, and it is also a potential prognostic marker.

## sHLA-G in Body Fluids of Tumor Patients as Tumor Markers

The source of sHLA-G is related to the inflammatory factors present in the cancer microenvironment and the reduction of NK cells and memory T cells (136, 137). Researchers have revealed that the expression of HLA-G in ascites, saliva, and bronchial lavage fluid can be useful for tumor diagnosis and prognosis (**Table 2**).

**Table 2:** Clinical research involving diagnosis and prognosis of HLA-G in the body fluid.

Cancer type	Sample source	Sample size	HLA-G type	Methods(Ab)	Experimental results evaluation	Expression evaluation of HLA-G	Ref.
**Breast cancer** **Ovarian cancer**	ascites specimens	24 (malignancy) 19 (negative)	sHLA-G	ELISA (W6/32)	The levels of sHLA-G were significantly higher in malignant compared with benign ascites	Measurement of sHLA-G is a useful molecular adjunct to cytology in the differential diagnosis of malignant *versus* benign ascite	(50)
**Ovarian cancer**	peritoneal fluid	16	sHLA-G1 HLA-G5	ELISA (MEM-G/9、5A6G7)	The level of HLA-G5 isoform was specifically higher in seven samples.	sHLA-G, particularly HLA-G5, may affect antitumor immune response both *in situ* and in circulation.	(39)
**Breast cancer**	effusions	46	HLA-G	IHC (4H84)	IHC showed predominantly focal HLA-G expression in 12 of 46 (26%) breast carcinoma effusions and 16 of 39 (41%) solid lesions	Associated with shorter disease-free survival.	(51)
**Ovarian cancer**	effusions	148	HLA-G	IHC (4H84)	HLA-G was detected in cancer cells in 49/148 (33%) effusions, 33/66 (50%) primary tumors, and 59/122 (48%) solid metastases.	A new role for HLA-G as a prognostic indicator in advanced-stage OC in effusions.	(47)
**Colorectal cancer**	saliva	20(patients) 10(controls)	sHLA-G1 HLA-G5	ELISA	In patients diagnosed with CRC, salivary sHLA-G values were significantly higher compared with the control group of healthy patients	sHLA-G can be a good prognostic and diagnostic biomarker in CRC.	(62)
**Colorectal cancer**	single-cell suspensions	157	HLA-G	Flow Cytometry Analysis (MEM-G/09)	The median percentage of HLA-G expression was 14.90% (range:1.81% to 79.90%).	HlA-G is closely associated with the survival of CRC patients.	(138)
**Oral squamous cell cancer**	saliva	22 (patients) 23 (controls)	sHLA-G	ELISA	There was no significant difference in shLA-G concentration between OSCC and control groups.	It helps tumor cells evade immune defense mechanisms.	(63)
**Lung cancer**	bronchoalveolar fluid	31	sHLA-G	ELISA	The mean value of soluble HLA-G was 49.04 ng/mL, and the level of HLA G varied greatly in metastatic tumors.	HLA-G soluble protein is significantly associated with patients with metastatic tumor and can be used as a prognostic marker of lung cancer.	(24)

## Ascites

Results obtained from Ullah’s group have shown that sHLA-G in ascites is mainly expressed by ascites cells, tumor cells, and stromal cells (39). Moreover, Sun et al. have found that the level of sHLA-G in malignant ascites induced by various solid tumors is significantly higher compared with benign ascites. Especially for ascites caused by gynecological tumors and gastrointestinal tumors, the levels of related tumor markers CEA and CA199 are also elevated in malignant ascites, while its specificity and sensitivity are significantly lower compared with sHLA-G (139). The level of sHLA-G in malignant ascites caused by OC and breast cancer is significantly higher compared with benign ascites. In addition, the specificity, sensitivity, and AUC are greatly improved when the critical value of 13 ng/mL is achieved. Besides, in OC, sHLA-G1/G5 is negatively correlated with CD3-/CD56+ subgroups and CD4+ CD45RO+ memory cells, suggesting that sHLA-G plays a role in the tumor microenvironment by up-regulating T-reg cells and down-regulating NK cells (39). In cytology-negative malignant ascites, sHLA-G also has outstanding diagnostic performance. In 32 cases of cytology-negative ascites, the positive rate of sHLA-G is 75%, which is significantly higher compared with CEA and CA199 (50). Collectively, sHLA-G can be used as an independent indicator for early diagnosis of malignant ascites, and it is helpful to screen for malignant ascites when cytology is negative.

## Saliva

Saliva is a body fluid that can be collected easily, quickly, and non-invasively (140). Biomolecules in saliva have been used as promising markers for early diagnosis, detection, and treatment in OSCC (141, 142), breast cancer (143, 144), OC (145), and other tumors. In OSCC, saliva is the body fluid that has the closest contact with oral tumors. Researchers have collected the saliva samples from 22 OSCC patients. Compared with non-metastatic OSCC, the HLA-G level of metastatic OSCC is dramatically elevated and correlated with poor OS (63). The sHLA-G level in CRC patients is also significantly higher compared with healthy controls, especially in patients with stage III-IV tumor (62). In the bronchial lavage fluid of patients with different histological types of lung cancer, the level of sHLA-G is significantly correlated with lower Karnofsky index in metastatic tumors and can be used as a prognostic marker for lung cancer (24). The exudate of OC patients is collected before, during, or after chemotherapy. It is found that the expression of HLA-G is decreased after chemotherapy, and the decreased expression of HLA-G predicts the improvement of patient survival rate. This may be related to the preferential sensitivity of HLA-G-expressing cells (47). Therefore, researchers believe that saliva sHLA-G can be used as a diagnostic and prognostic biomarker for multiple cancers, and it is a less invasive alternative to venipuncture. However, more studies are needed to confirm the significance of saliva sHLA-G as a tumor indicator.

## HLA-G Derived From EVs as a Tumor Marker

EVs are composed of growth factors, biologically active lipids, genetic information, and antibodies/ligands/receptors, and they are resistant to RNase, enduing their great potential as a tumor biomarker (146–148). Secreted HLA-G can exist in the form of free sHLA-G or be secreted by EVs and found in various body fluids, such as plasma, ascites, and pleural exudate (96) (**Figure 2**). Recent studies have found that tumor cells, cytotrophoblast cells, and mesenchymal stem cells can secrete HLA-G-carrying EVs, playing a role in regulating the tumor microenvironment and immunosuppressive function (149). Therefore, HLA-G can be shedding from the cell surface by metalloproteases or released from various cells, incorporated into EVs, and serve as a promising tumor indicator (77, 104, 150).

Riteau et al. have isolated an HLA-G-positive cell line from primary and metastatic lymph node melanomas and named it Fon. For the first time, this melanoma cell line is found to secrete exosomes containing HLA-G1. They speculate that the immune tolerance produced by melanoma-derived HLA-G exosomes may be a method for tumors to regulate host immunity. So far, the specific mechanism of this method is still unclear (150). In breast cancer patients receiving neoadjuvant chemotherapy (NACT), the relationship between exosomes carrying HLA-G and the prognosis of the disease has been evaluated. Before NACT, the sHLA-G_EV_ levels are correlated with circulating stem cell-like tumor cells. The total amount of sHLA-G_EV_ is significantly increased after NACT, and it is related to the disease process, while the total sHLA-G level has nothing to do with the clinical prognosis (48). In addition, HLA-G-bearing EVs released by renal cancer cells damage the differentiation of monocytes into DCs and inhibit the maturation process of DCs. These findings suggest that HLA-G-mediated tumor immune escape mechanisms can spread to HLA-G-negative tumor cells through the EV pathway (151, 152). In epithelial ovarian cancer (EOC), the levels of HLA-G_EV_ are increased by seven times compared with healthy controls, and the elevated HLA-G_EV_ can serve as independent 3-year and 10-year progression-free survival (PFS) prognostic factors. It is worth noting that all patients with high levels of HLA-G_EV_ experience disease progression within approximately 5 years after the initial diagnosis. Schwich’s group has also shown that HLA-G_EV_ serves as an independent risk assessment marker for disease progression of EOC (40).

## HLA-G Detection and Quantification

Based on the current understanding of HLA-G and its various forms that have multiple immune tolerance regulating functions in malignant tumors, HLA-G is generally recognized as a biomarker that can be used to monitor the disease state and progression in cancer patients (153). However, due to the diversity of HLA-G structures, the standardization of HLA-G detection methods has been a topic of discussion from the past to the present (154). HLA-G test results vary greatly in different locations of HLA-G acquisition, between different tumors, or the same tumor in different laboratory test results.

## Detection of HLA-G in Tissues

The status of HLA-G in tumor tissues is usually detected by IHC. Although IHC is an experimental technique that has been widely used, there is controversy about the use of such a method to detect HLA-G. Because HLA-G has multiple subtypes, and each antibody recognizes only specific epitopes, leading to different staining results (52, 54, 124, 155). In addition, the experimental procedures of IHC also differ greatly, such as the type of antibody used and its dilution, incubation time, and staining evaluation criteria. The differences in treatment history, tumor subtypes, and individual tumor microenvironment between different patients will also affect the evaluation of HLA-G expression levels (156–158).

In 81 patients with colon cancer, Swets and colleagues have used three monoclonal antibodies (4H84, MEM-G/1, and MEM-G/2) to evaluate the expression of HLA-G. In primary tumors, the positive staining rates of HLA-G using monoclonal antibody 4H84, MEM-G/1, and MEM-G/2 are 29%, 6%, and 10%, respectively. They have found that different epitopes of HLA-G detected by different monoclonal antibodies are differentially expressed in CRC tissues (159–161). Although it has been confirmed that 4H84 and MEM-G/1 can recognize all subtypes of HLA-G, the reason for this difference in expression is generally believed to be a cross-reaction with HLA-I (160, 161). For example, 4H84 has been shown to cross-react with the existence of β2M free classic HLA class I molecules on activated leukocytes. This may lead to an overestimation of HLA-G expression in pathological tissues recognized by leukocyte infiltration (such as CRC), leading to differences among studies (162). Therefore, it is recommended to use a variety of different monoclonal antibodies to detect HLA-G.

In addition, whether these antibodies can block the binding of HLA-G to its receptors (ILT2, ILT4, and KIR2DL4) is also a challenge (163). Antibodies targeting the α3 domain and β2M of HLA-G can block the binding of HLA-G to ILT2 and ILT4. The antibody targets the α2 domain and can block the binding of HLA-G to KIR2DL4. It has been confirmed that MEM-G/1 blocks the binding site of HLA-G2 and ILT4, while MEM-G/9 and G233 can bind to HLA-G1, depriving the binding site of ILT2. Besides, antibody 87G can block the interaction between HLA-G1 and its receptor (164). Therefore, to block the interaction of HLA-G with all its receptors, it is necessary to develop an antibody mixture that can recognize all HLA-G subtypes and block the binding of HLA-G to ILT2, ILT4, and KIR2DL4, thereby reducing HLA-G cross-reaction with its receptors.

## Detection of sHLA-G in Fluids

sHLA-G is also expressed and released by cancer cells, which is a potential biomarker in the body fluids of cancer patients (62). In particular, sHLA-G in the supernatant of IVF embryos has been considered as an independent factor predicting pregnancy outcome (165–167). Therefore, as early as 2004, in the Wet Workshop for the quantification of sHLA-G, the standardization of sHLA-G detection and quantification methods has been discussed, including sensitivity, standard reagents, and antibody specificity (168). At present, ELISA is mainly employed to quantify the level of sHLA-G in body fluids. The monoclonal antibody MEM-G/9 is used to simultaneously detect the shedding of HLA-G1 and the secreted HLA-G5. MEM-G/9 specifically captures β2M-related HLA-G1 and HLA-G5 as well as polyclonal anti-human β2M antibodies (169). In this seminar, HLA-G-expressing EVs are also discovered. This structure cannot be detected by the combination of MEM-G/9 and anti-β2 but can be detected by an antibody combination of mAb 5A6G7 and W6/32. Given the often-antagonistic composition and complex structure of EVs, the various components carried by EVs may drive the functional activity of EVs expressing HLA-G and eliminate or enhance the immune tolerance function of HLA-G.

ExoQuick™ precipitation is now usually used to extract EVs from samples and to quantify the number of EV particles and the number of vesicular-bound HLA-G (HLA-G_EV)_ (48). The results show that in OC, poor clinical status and presence of CTC, and PFS are associated with elevated HLA-G_EV_ levels (40). In breast cancer patients receiving NACT, high levels of vesicular sHLA-G are also associated with disease progression (48). Given these, future work should focus on the standardization process. Before being applied to routine clinical practice, a larger research cohort, prospective research, and internationally recommended standardized testing methods are needed to verify the application of HLA-G in diseases. Importantly, it is necessary to develop more specific antibodies against HLA-G subtypes, explore new undiscovered HLA-G subtypes, and make full use of existing experimental techniques to evaluate the role of various subtypes in various tumors.

## Conclusions

After more than 30 years of research, it has been shown that compared with other tumor markers, HLA-G has unique characteristics as follows: 1) it participates in the immune tolerance network in healthy individuals and tumor patients; 2) it is barely expressed in normal tissues and frequently identified in tumor cells. Given these characteristics of HLA-G, one of the current research goals is to serve as a biomarker for diagnosis, prognosis, and clinical testing. However, many factors affect the interpretation of the clinical significance of HLA-G, such as the number of patients included in the study, different clinical parameters of patients (such as medical history, disease stage, and treatment plan), and differences in testing protocols. To better explain the structural diversity of HLA-G, sHLA-G, HLA-G_EV_, and their expression and clinical significance in tumors, future efforts should be devoted to studies focusing on multi-center and large sample analysis, and the establishment of standardized and feasible HLA-G detection methods.

## Author Contributions

PL, NW and YZ wrote and edited the manuscript. CW and LD edited the manuscript. All authors contributed to the article and approved the submitted version.

## Funding

This research was supported by grant from the National Natural Science Foundation of China (82002228 to PL, 82072368 to LD), the Key Research and Development Program of Shandong Province (2019GSF108091 to LD), Taishan Scholars Program of Shandong Province awarded to CXW and LD.

## Conflict of Interest

The authors declare that the research was conducted in the absence of any commercial or financial relationships that could be construed as a potential conflict of interest.

## Publisher’s Note

All claims expressed in this article are solely those of the authors and do not necessarily represent those of their affiliated organizations, or those of the publisher, the editors and the reviewers. Any product that may be evaluated in this article, or claim that may be made by its manufacturer, is not guaranteed or endorsed by the publisher.
